# Inhibition of cancer antioxidant defense by natural compounds

**DOI:** 10.18632/oncotarget.13723

**Published:** 2016-11-30

**Authors:** Alicja Sznarkowska, Anna Kostecka, Katarzyna Meller, Krzysztof Piotr Bielawski

**Affiliations:** ^1^ Department of Biotechnology, Intercollegiate Faculty of Biotechnology, University of Gdansk and Medical University of Gdansk, Gdansk, Poland

**Keywords:** antioxidants, ROS, cancer, natural compounds, polyphenols

## Abstract

All classic, non-surgical anticancer approaches like chemotherapy, radiotherapy or photodynamic therapy kill cancer cells by inducing severe oxidative stress. Even tough chemo- and radiotherapy are still a gold standard in cancer treatment, the identification of non-toxic compounds that enhance their selectivity, would allow for lowering their doses, reduce side effects and risk of second cancers. Many natural products have the ability to sensitize cancer cells to oxidative stress induced by chemo- and radiotherapy by limiting antioxidant capacity of cancer cells. Blocking antioxidant defense in tumors decreases their ability to balance oxidative insult and results in cell death. Though one should bear in mind that the same natural compound often exerts both anti-oxidant and pro-oxidant properties, depending on concentration used, cell type, exposure time and environmental conditions. Here we present a comprehensive overview of natural products that inhibit major antioxidant defense mechanisms in cancer cells and discuss their potential in clinical application.

## INTRODUCTION

Over 60% of currently used antitumor drugs come from natural sources such as plants, fungi and microorganisms. The large scale screening programs for natural products with anticancer activities, e.g. those launched in 1950s by Italian research company or in 1960s by the National Cancer Institute (NCI), allowed for identification of bacteria-produced doxorubicin and taxol (paclitaxel), derived from the bark of the yew tree. Both of these compounds are widely used in chemotherapy regimens in different cancer types. Though their mechanism of action is different as doxorubicin intercalates into DNA and abrogates replication [1] and taxol inhibits microtubules depolymerization during mitosis [2], they both induce strong oxidative stress, though by different means [3–5]. Total cellular antioxidant capacity is a known determinant of cancer susceptibility to these drugs [6–8]. Oxidative stress induced by chemotherapeutics is crucial for their efficacy, but, on the other hand, contributes to the cumulative and irreversible cardiotoxicity observed clinically [9, 10]. These side effects highlight the lack of selectivity of chemotherapy [11]. Therefore, non-toxic natural substances that potentiate action of chemotherapeutics and allow for lowering their concentration are of a particular interest to the anticancer drug field.

## ROS IN CELLULAR TRANSFORMATION

**Figure 1:**
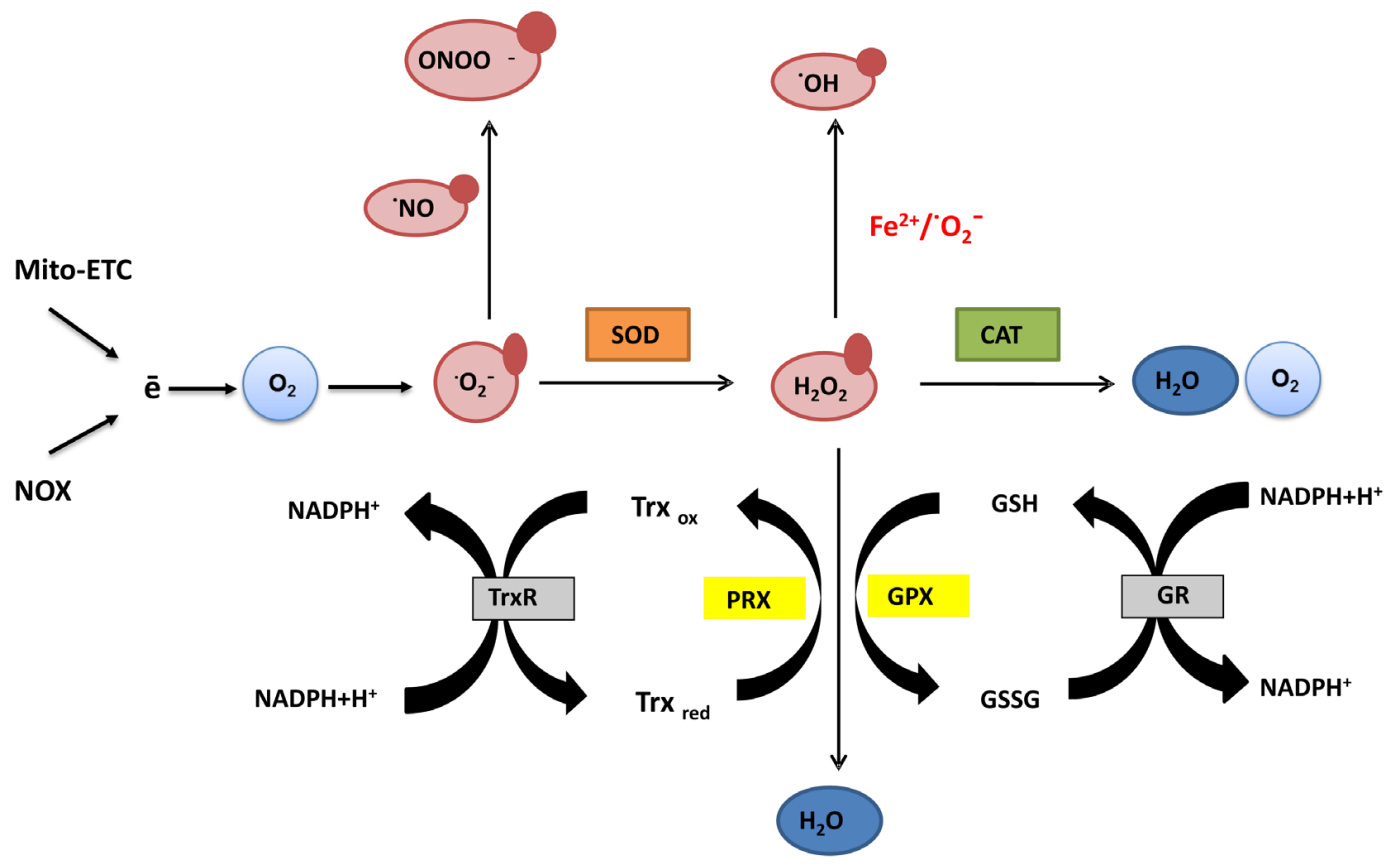
Generation and scavenging of reactive oxygen species (ROS) Electrons released from the mitochondrial electron transport chain (Mito-ETC) and produced by NADPH oxidases (NOX) are the major source of endogenous reactive oxygen species. Coupled to molecular oxygen they give rise to the primary free radical and the precursor of remaining species - superoxide (·O_2_^-^). In the reaction with a short-lived nitric oxide (·NO), superoxide forms a highly reactive peroxynitrate (ONOO^-^) able to modify structure and function of proteins. Alternatively, superoxide dismutase (SOD) converts superoxide to hydrogen peroxide (H_2_O_2_), which can be further transformed in several ways. In the presence of transition metal ions like Fe^2+^ (Fenton's reaction) or in reaction with superoxide, H_2_O_2_ forms highly reactive hydroxyl radical (·OH) which damages lipids, proteins and DNA. Peroxysomal enzyme catalase (CAT) neutralizes H_2_O_2_ to water and oxygen. H_2_O_2_ might be also utilized in the reaction of oxidation of monomeric glutathione (GSH) to the glutathione disulfide (GSSG) or reduced thioredoxin (Trx_red_) to the oxidized thioredoxin (Trx_ox_) catalyzed by glutathione peroxidase (GPX) or peroxidases involved in the thioredoxin turnover (PRX). Reduced glutathione pool is restored by glutathione reductase (GR) which reduces oxidized glutathione with the use of NADPH. Similarly, thioredoxin reductase (TrxR) balances the amount of reduced Trx by transferring electrons from NADPH to oxidized catalytic sites. Thanks to the thiol groups in the Cys residues both glutathione and thioredoxin participate in the reduction of oxidized proteins. Their synthesis as well as the turnover are under tight homeostatic control creating a system responsible for reduction of redox-sensitive proteins upon oxidative stress.

Majority of cellular reactive oxygen species (ROS) is produced during aerobic respiration by electrons released from the electron transport chain (ETC) in mitochondria. Incomplete oxygen reduction creates superoxide anion (O_2_.^-^), the precursor of three remaining species: hydroxyl radical (OH.), hydrogen peroxide (H_2_O_2_) and peroxynitrite (OONO-) [12] (Figure 1). Mitochondrial electron leakage increases with age pointing to the imbalance between mitochondrial biogenesis and degradation - a root cause of neurodegenerative and cardiovascular diseases, diabetes and cancer [13]. The second largest contributor to cellular ROS are NADPH oxidases (NOX) residing in cytoplasm, catalyzing the production of superoxide from O_2_ and NADPH [14, 15]. At low concentrations, superoxide production may be involved in cellular signal transduction, but high concentrations of radicals cause oxidative damage due to their high reactivity towards other cellular compounds [16].

Higher steady-state levels of ROS in cancer cells relative to normal cells have been known for around 35 years [17]. Increased ROS are crucial in the initiation of carcinogenesis when acquiring new mutations and clonal expansion of initiated cells are needed to establish a tumor. This renders them both the cause and the result of cellular transformation: ROS-induced oxidative damage favors production of more radicals and establishes a feed-in loop, increasing mutations rate, activating oncogenes, enhancing metabolic reprogramming and progression of tumors. The enhanced ROS generation is induced by oncogenic signaling with main drivers: V-Ras, K-Ras, mtp53 and c-Myc [18, 19] and involves both mitochondrial and cytoplasmic ROS. K-Ras-induced cellular transformation was shown to require NOX1 activation through p38/PDPK1/PKCδ/p47phox cascade [20], while expression of Myr-Akt, H-RasG12V and K-RasG12D in murine embryonic fibroblasts (MEFs) conferred increased mitochondrial ROS-dependent soft agar colony formation [21]. Mutations in tumor suppressors genes are often associated with the induction of strong oxidative stress and promote the survival of cells with high ROS levels. Mutant BRCA1 and p53 were shown to attenuate antioxidant signaling driven by the nuclear factor (erythroid-derived 2)-like 2 (Nrf2), contributing to cancer initiation [22, 23]

One of the consequences of the excessive damage caused by ROS are changes in mitochondrial membrane permeability that result in cytochrome C release and apoptotic death [24–29]. In defense, cancer cells boost their antiapoptotic mechanisms like nuclear factor kappa-light-chain-enhancer of activated B cells (NFĸB) pathway to escape cell death [30, 31]. Decreased mitochondrial activity triggers the glycolytic switch and upregulates glycolytic pathway in order to produce more energy and biomass (ribose, amino acids, fatty acids) for rapidly proliferating cancer cells [32]. Moreover, exposure to oxidative stress induces mutations in mitochondrial DNA as well as in *VEGF* (Vascular Endothelial Growth Factor) and *HIF-1α* (Hypoxia Inducible Factor-1α) genes, promoting angiogenesis and further enhancing metabolic reprogramming of cells [33]. Oxidative stress also changes the tumor microenvironment to support growth and cell spread. Hydrogen peroxide produced by tumor tissue can initiate destruction of non-tumor surrounding tissue to obtain nutrients and promote growth [34]. This explains why tumors are said to be “addicted to ROS signaling”.

## ROS ADAPTATIONS IN TUMORS

Distinct redox homeostasis and higher intracellular ROS levels in cancer cells drive their growth and metastasis but might also pose a threat of oxidative damage and death. Moderate expression of NADPH oxidase NOX5-L induced cancer cells proliferation accompanied by AKT and ERK phosphorylation, whereas an increase in NOX5-L above a certain threshold promoted apoptosis [35]. Tumors need to adapt to the oxidative stress conditions and they do that by enhancing their antioxidative defense to lower ROS levels and by inducing autophagy to reduce the oxidative damage to biomolecules and organelles [36–39]. These two mechanisms constitute finely orchestrated and interconnected repair system in oxidatively stressed cells seeking homeostasis [36]. Interestingly, the same oncogene signals that boost ROS signaling, promote antioxidant adaptive mechanisms to stand this constant stress and minimize oxidative damage. Activation of endogenous K-Ras(G12D), B-Raf(V619E) and Myc(ERT2) led to lowering of intracellular ROS due to the increased transcription of Nrf2 and elevation of the basal Nrf2 antioxidant program [40]. Furthermore, genetic targeting of the Nrf2 pathway impaired K-Ras(G12D)-induced proliferation and tumorigenesis *in vivo* pointing that the Nrf2 pathway represents a previously unappreciated mediator of oncogenesis [40]. Accordingly, it was reported that genetic mutations that occur in cancer cells led to constant Nrf2 activity and enhanced antioxidant capacity [41]. Harris et al. (2015) showed that synthesis of the antioxidant glutathione (GSH) was required for cancer initiation *in vivo* [42]. Genetic loss of the enzyme driving GSH synthesis, glutamate-cysteine ligase modifier subunit (GCLM), prevented a tumor's ability to drive malignant transformation. Interestingly, at later stages of tumor progression GSH became dispensable potentially due to the compensation from an alternative antioxidant pathway - thioredoxin pathway, demonstrating the importance of GSH and thioredoxin to tumor progression and indicating them as potential targets for therapeutic intervention.

Mitochondrial ROS are the major inducers of autophagy, however, upon chronic impairment of mitochondrial function, high extent of radicals shifts signaling into self-removal of mitochondria through a selective process called mitophagy [43, 44]. This fine mechanism allows autophagy to eliminate the source of oxidative stress and protect the cell from oxidative damage. Recently, autophagy was shown to prevent the initiation of hepatocarcinogenesis and metastasis of gastric cancer by maintaining healthy mitochondria and reducing oxidative stress and DNA damage [45–47]. On the other hand, once the cellular transformation was initiated, autophagy was required to promote cancer progression by limiting tumor suppressors [45].

## TARGETING ROS ADAPTATIONS IN CANCER

Because of this sharp reliance on ROS production, cancer cells are more vulnerable to further disturbance of their red-ox status than normal cells. This difference establishes a therapeutic window allowing for an emergence of the selective anticancer strategy based on modulation of cancer cells redox potential. Due to the enhanced antioxidant capacity of tumors, just inducing ROS generation is not sufficient for a successful eradication of cancer. The drug should also inhibit the antioxidant defense system [48]. Many compounds of natural origin block Nrf2 pathway or directly inhibit endogenous antioxidants leading to the elevated ROS production. Moreover, Nrf2 inhibition results in a decrease of drug efflux transporters and a consequent increase in retention of anticancer drugs in cells. Therefore Nrf2 or cellular antioxidant inhibitors synergize with classic chemotherapeutics and decrease their toxicity. Surprisingly, among them there are polyphenols like resveratrol, quercetin, EGCG, apigenin, luteolin or chrysin which were initially reported to have ROS scavenging properties and are generally recognized as antioxidants. Therefore a considerable caution should be exercised when applying natural products as adjuvants since their effects strongly depend on concentration, cell type, exposure time and environmental conditions [49–55].

## THE NRF2 PATHWAY

**Figure 2:**
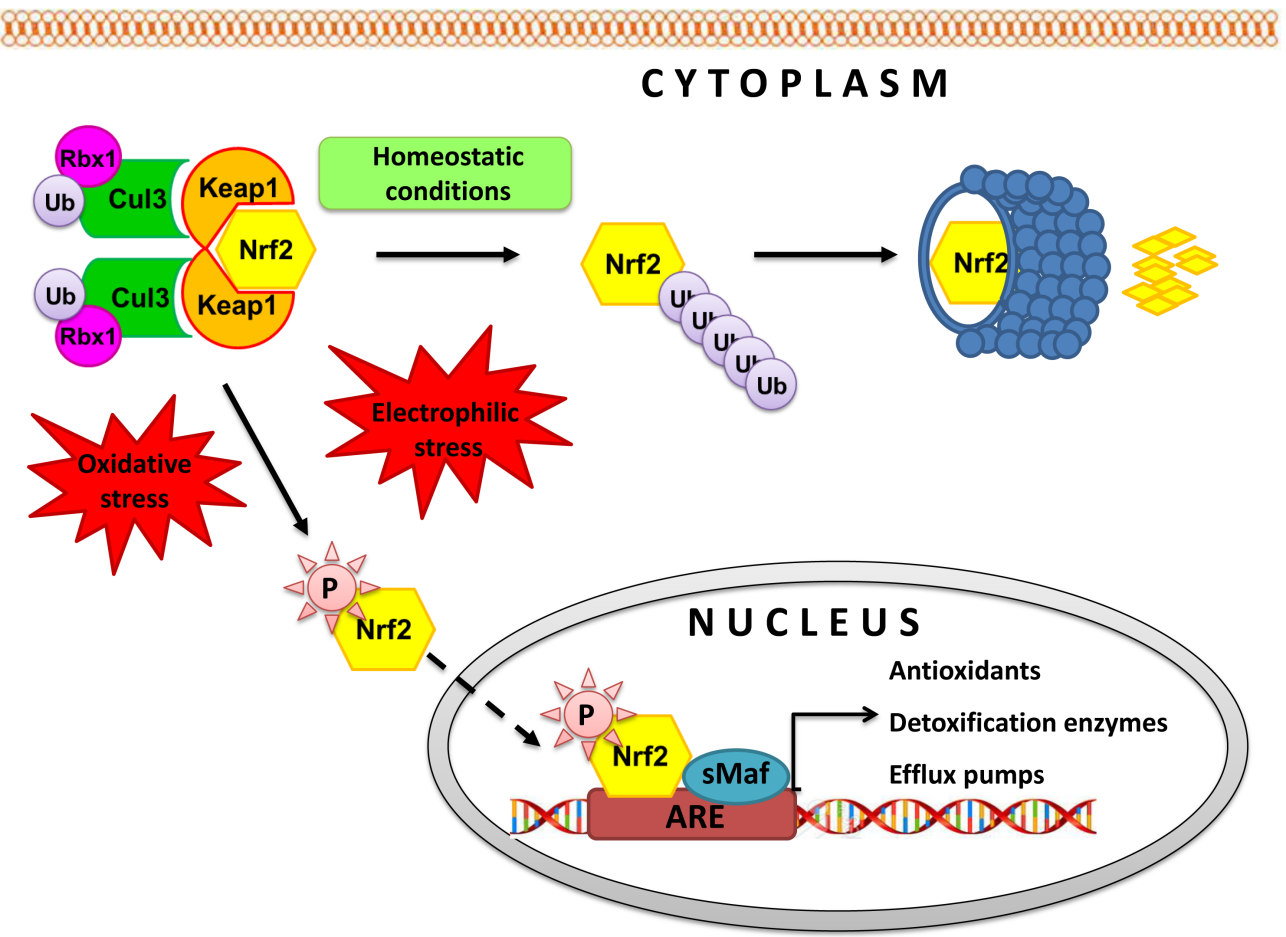
Regulation of Keap1-Nrf2 pathway Under basal conditions, cytosolic repressor Kelch-like ECH-associated protein 1 (Keap1), a substrate adaptor protein for Cullin 3 (Cul3)/Rbx1 ubiquitin ligase, holds Nrf2 in the cytoplasm and promotes its ubiquitination followed by 26S proteasomal degradation [58,59]. In the presence of electrophilic and/or oxidative stimulus, Nrf2 is released from Keap1 and translocates to the nucleus where it recruits small Maf protein (sMaf) and binds with response element (ARE) in the promoter regions of its target genes, inducing their expression. Activation of Nrf2 pathway allows for cell adaptation and survival by regulating expression of antioxidans, anti-inflammatory and phase II detoxification enzymes such as superoxide dismutase (SOD), gluthatione S-transferase (GST), heme oxygenase-1 (HO-1), NAD(P)H-quinone oxidoreductase (NQO1), UDP-glucuronosyl transferases (UGT), γ-glutamylcysteine synthetase (γGCS) and efflux pumps like multidrug resistance-associated protein 2 (MRP2) and breast cancer resistance protein (BCRP). Proteins transcriptionally controlled by Nrf2 take part in biosynthesis, utilization and regeneration of glutathione, thioredoxin, and NADPH resulting in restoration of cellular redox homeostasis.

**Figure 3:**
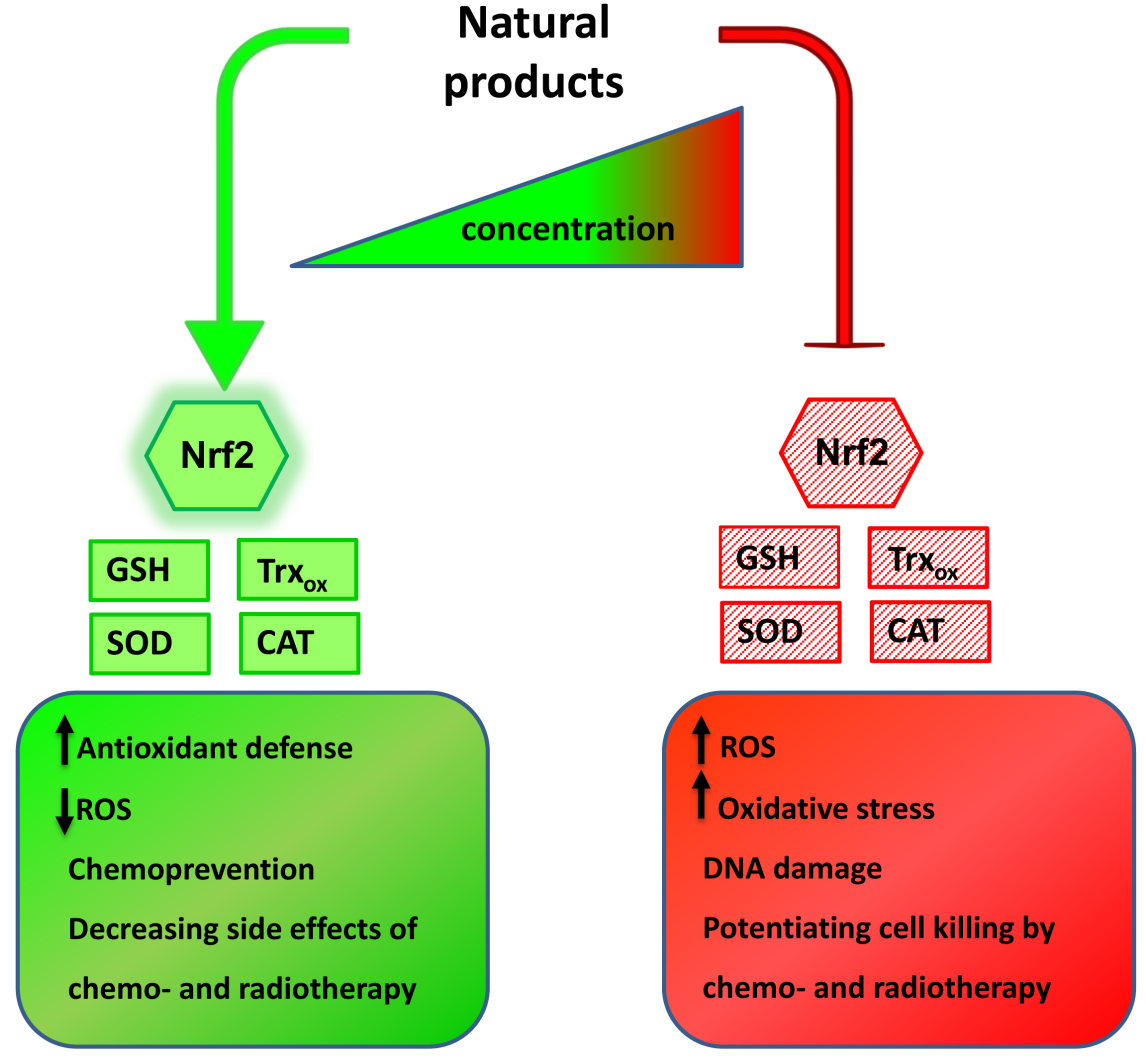
Natural products action on cellular antioxidants is concentration-dependent Many natural compounds display opposing properties in cancer cells, depending on their concentration. At lower concentrations they often boost cells’ antioxidant capacity by activating Nrf2-dependent signaling and enhancing expression of ROS scavengers, lowering ROS burden. These properties allow for using natural compounds in chemoprevention and as agents decreasing side effects of standard anticancer regimens. On the other hand, same compounds used at higher concentrations inhibit antioxidant defense and induce oxidative stress. By doing that they enhance the effectiveness of chemo- and radiotherapy and allow for lowering their doses.

Disruption of redox balance in cells results in activation of redox sensitive transcription factors like Nrf2, NFĸB and activator protein 1 (AP-1) [56]. The major driver of antioxidants expression that confers protection against endogenous and exogenous hazards, DNA damage and consequent cancer initiation is Nrf2 transcription factor [41, 57]. Activation of Nrf2 pathway allows for cell adaptation and survival by regulating expression of antioxidans, anti-inflammatory and phase II detoxification enzymes (Figure 2). Major regulator of Nrf2 activity in cells is the cytosolic inhibitor Keap1, responsible for its ubiquitination and proteasomal degradation [58, 59]. Apart from Keap 1, oncogenes like K-Ras(G12D), B-Raf(V619E) and Myc(ERT2) have been shown to stabilize Nrf2 and antioxidant proteins leading to drug resistance in tumors [40]. Nrf2 is overexpressed in several types of human cancer, including cancer of the lung, breast, oesophagus, ovary, prostate, pancreatic, colorectal, head and neck squamous cell carcinoma, gallbladder and skin which indicates that the cytoprotective properties of the Nrf2 pathway can be exploited by tumor cells to promote their survival [60, 61]. Constitutive Nrf2 activation has been reported to mediate chemoresistance in many tumor types [62, 63]. Suppression of Nrf2 activity inhibited tumor growth and enhanced the efficacy of chemotherapeutic agents. Disruption of the Nrf2 pathway in a mouse model of K-RasG12D-induced lung cancer enhanced the antitumor efficacy of cisplatin [64]. Temporal blockage of Nrf2-dependent cytoprotection using Nrf2 inhibitors is important to enhance a patient's response to chemo- and radiotherapy but on the other hand, activation of Nrf2 pathway supports treatment of neurodegenerative diseases, multiple sclerosis and prevents cancer initiation by counteracting oxidative and electrophilic stress [60]. It means that in case of cancer, the Nrf2 pathway is a double edge sword: activating this pathway is crucial for chemoprevention but once the control is lost, it provides growth advantage to cancer cells allowing for rapid proliferation, escape from apoptosis or senescence and resistance to chemo- and radiotherapy. Thus, both activation and inhibition of Nrf2 activity could be beneficial, although in different patient cohorts (Figure 3).

## NATURAL PRODUCTS TARGETING NRF2 PATHWAY

Natural product-derived inhibitors of Nrf2 pathway induce ROS insult in ROS-sensitive cancer cells which might result in cell death. Importantly, they often sensitize cancers to the effects of chemotherapeutics or radiotherapy through the down-regulation of detoxification enzymes and drug excretion transporters [65, 66]. A significant group of Nrf2 inhibitors belongs to polyphenols (see Table 1). Polyphenols are generally recognised as antioxidants and anti-inflammatory agents. At low to micromolar concentrations polyphenols like quercetin, EGCG, resveratrol or curcumin exhibit antioxidant and chemopreventive properties. They can scavenge free radicals either directly, due to the presence of OH groups donating a hydrogen atom to a free radical, or by indirect actions through the induction of Nrf2 pathway or inhibition of ROS generation. Higher doses of polyphenols (>50 µM) and a presence of transition metal ions promote their pro-oxidant actions like suppression of antioxiant systems and inhibition of Nrf2 pathway [49]. Antitumor effects of flavones like apigenin, chrysin, luteolin and wogonin was related to the downregulation of Nrf2 expression mainly by disturbing PI3K/Akt pathway in cell lines and in *in vivo* mouse models. Nrf2 inhibition sensitized cancer cells to classic chemotherapeutic drugs like doxorubicin, oxaliplatin or paclitaxel both in *in vitro* and *in vivo* studies [67–70]. Interestingly, also opposite activity of apigenin, luteolin and chrysin was reported. In rat primary hepatocytes and skin epidermal JB6 P+ cells these flavones induced Nrf2/ARE response and protected against oxidative stress [71, 72]. Differences in their activity between normal and cancer cells and encourage further investigation of their potential in *in vivo* studies and clinical trials. So far, none of these flavones has been tested clinically for the anticancer activity in combination with chemo- or radiotherapy. Brusatol, a triterpenoid from *Brucea javanica* - an evergreen shrub grown in Southeast Asia and Northern Australia, was described to inhibit Nrf2 signaling by enhancing ubiquitination and subsequent degradation of Nrf2 in different cancer cell lines and mouse xenograft models [73]. Brusatol sensitized tumors to cisplatin and taxol [73–75]. The bitter coffee alkaloid, trigonelline, inhibited nuclear accumulation of Nrf2 in pancreatic cell lines (Panc1, Colo357 and MiaPaca2) and H6c7 pancreatic duct cells and enhanced their sensitivity to anticancer drugs and TRAIL-induced apoptosis [76]. A naphthoquinone derived from *Plumbago* species, plumbagin, inhibited nuclear translocation of Nrf2 in human tongue squamous cell carcinoma cells which suppressed the expression of Nrf2 downstream targets resulting in inhibition of epidermal to mesenchymal transition (EMT) and stemness [77]. Parthenolide, a sesquiterpene lactone found in feverfew products, was recently reported to inhibit Nrf2 protein level in breast cancer stem-like cells, derived from dissociation of mammospheres which correlated with an increased ROS production and led to necrosis [78].

**Table 1:** Natural products inhibiting antioxidant capacity of cancer cells

BIOACTIVE COMPOUND	TYPE	SOURCE	MECHANISM OF ACTION
Apigenin	Polyphenol Flavonoid Flavone	Fruits and vegetables	Reduces Nrf2 expression through down-regulation of PI3K/Akt pathway [67] Sensitizes tumor xenografts to doxorubicin [67] Induces glutathione depletion [94] and inhibits mitochondrial complex I activity in rats [151]
Chaetocin	Polyphenol thiodioxopiperazine	*Chaetomium spp.* fungi	Inhibits TrxR *in vitro* [117]*;* induces oxidative stress-mediated death of myeloma [152] and glioma cells [118]
Chrysin	Polyphenol Flavonoid Flavone	Passion flowers, chamomile, honeycombs, oyster mushrooms	Reduces Nrf2 expression in hepatocellular carcinoma through down-regulation of PI3K-Akt and ERK pathways re-sensitizing cells to doxorubicin [153] Depletes glutathione and enhances doxorubicin-induced cytotoxicity in epithelial cancer cells [69,94]
Curcumin	Polyphenol Curcuminoid	Rhizomes of *Curcuma longa*	Inhibits TrxR required for curcumin-induced radiosensitization [107,119] Inhibits NF*κ*B signaling in different cancer types [154–157]
Epigallocatechin gallate (EGCG)	Polyphenol Flavonoid Falvon-3-ol Catechin	White, green and black tea (buds and leaves of *Camellia sinensis)*	Inhibits TrxR and induces cancer cells death [158] Inhibits catalase, leads to elevated ROS [129] Degrades catalase via JNK in endothelial cells [159] Synergize with luteolin to induce apoptosis and p53 activation in cancer cells, reducing growth of xenografts [113]
Luteolin	Polyphenol Flavonoid Flavonol	Celery, green pepper, parsley, perilla leaf, and chamomile tea	Reduces Nrf2 expression in non-small-cell lung cancer cells, leading to GSH depletion [160] Sensitizes cells to oxaliplatin, bleomycin, doxorubicin [160,161]. Re-sensitizes oxaliplatin-resistant colorectal cancer cells [68] Inhibits Nrf2 in xenografts [162]
Myricetin	Polyphenol Flavonoid Flavonol	*Citrus spp*.	Blocks GST activity in melanoma cells [160] Inhibits TrxR leading to death of lung carcinomas [114]
Quercetin	Polyphenol Flavonoid Flavonol	*Citrus spp*.	Inhibits TrxR leading to death of lung carcinomas [114] Inhibits mitochondrial complex I activity in rats [151]
Resveratrol	Polyphenol Stilbenoid	grapes, raspberries, blueberries, mulberries	Directly binds and inhibits NQO2 and GSTP1 [163–165] Blocks mitochondrial I and III complex activity in colon cancer [166]
Wogonin	Polyphenol O-methylated flavone	roots of *Scutellaria baicalensis Georgi*	Down-regulates Nrf2 in resistant myelogenous leukemia cells by modulating PI3K/Akt and DNA-PKcs [167] Inhibits catalase, increasing H2O2. Synergistically sensitizes cancer cells derived from cervix, ovary and lung to TNF-induced apoptosis by blocking TNF-induced NF-κB activation [127]
Brusatol	Alkaloid Triterpenoid Quassinoid	*Brucea javanica*	Reduces Nrf2 via Nrf2 ubiquitination and degradation [73] Sensitizes xenografts to cisplatin via Nrf2 inhibition [73] Down-regulates Nrf2, leading to ROS accumulation. Sensitizes mammospheres to taxol and reduces the anchorage-independent growth [75] Inhibits Nrf2 in freshly isolated primary human hepatocytes [74] Enhances efficacy of cisplatin [64]
Piperlongumine	Alkaloid Pyridine group	Fruits and roots of long pepper	Binds GSH and inhibits its metabolism in leukemias [92] Increases I*κ*B*α* and suppresses NF*κ*B in human gliomas resulting in ROS-induced apoptosis [93]
Trigonelline	Alkaloid Pyridine and piperidine group	coffee	Reduces nuclear accumulation of Nrf2 in pancreatic cancer cells and sensitizes them to anticancer drugs and TRAIL *via* Nrf2 inhibition [76]. Enhances response to chemotherapy *in vivo* [76]
Pentyl isothiocyanate (PEITC)	Glucoside Glucosinolate	Cruciferous vegetables	Reacts with glutathione; lowers GSH [168]; inhibits GPX, depletes GSH, disrupts GSSG/GSH ratio [169,170] Decreases SOD in gliomas [171] Inhibits mitochondrial respiratory chain I in leukemias [172]
Pleurotin	Quinone	mushrooms from *Pleurotus spp*.,	Inhibits TrxR in breast cancer and colon carcinoma lines, leading to HIF-1α downregulation and growth inhibition [173,174]
Allicin	Organosulfur compound	garlic	Induces GSH depletion in pancreatic cancer cells [96] Inhibits NFκB signaling activation [175]
Plumbagin	Naphthoquinone	*Plumbago sp*.	Inhibits Nrf2 signaling in human squamous carcinoma cells [77] Depletes intracellular GSH level and SOD2 in prostate cancer cells [97] Inhibits NFκB activation in human non-small lung cancer cells [176], pancreatic [177] and gastric cancer cells [178]
EM23	Terpene Sesquiterpene lactone	*Elephantopus mollis*	Attenuates TrxR by alkylation of C-terminal redox-active site Ser498; inhibits Trx/TrxR expression facilitating ROS accumulation in human cervical cancer cells [179] Suppresses TNF-α-mediated activation of NFκB in CML cells and AML leukemia cells[180]
Parthenolide	Terpene Sesquiterpene lactone	*Tanacetum parthenium*	Downregulates Nrf2 expression in spheroids cultures [78] Activates NADPH oxidase, decreasing reduced thioredoxin and activating PI3K/Akt, inducing FOXO3a phosphorylation and resulting in downregulation of FOXO3a-regulated antioxidants (SOD, CAT) [78] Inhibits NFκB activity by binding and suppressing IκB kinase β [180]

## THE CELLULAR ANTIOXIDANT DEFENSE

Increased levels of free radicals enable tumor cells to activate pathways driving proliferation, angiogenesis, metastasis and thrive under hypoxic conditions [79–81]. High levels of ROS create the risk of damage linked to oxidative stress, therefore cancer cells tend to overexpress detoxifying proteins that elevate their antioxidant capacity. Hyper-activation of Nrf2 pathway increases the amount of cellular ROS scavengers. Lowering stress burden by means of enhancing detoxifying force further affects certain pathways that promote growth and proliferation [82–84]. Blocking antioxidant activity in cancer cells decreases their ability to balance oxidative insult and might result in cell death [85]. Below are presented key cellular antioxidant systems and natural compounds disturbing their activity

## GSH

One of the major systems involved in response to free radicals relies on a tripeptide - glutathione. The sulfhydryl (SH) group of reduced glutathione accounts for its strong electron-donating properties (Figure 1). Once oxidized, two glutathione molecules form a dimer linked by a disulfide bridge (GSSG). GSH reacts with proteins to form S-glutathionylated proteins, protecting them from further oxidation. Glutathione not only directly scavenges free radicals (hydroxyl radical, singlet oxygen), but also serves as a cofactor of several detoxifying enzymes that require thiol-reducing equivalents (glutathione peroxidase, glutathione transferase). GSH is also involved in recycling other antioxidants by reducing vitamins C and E [86]. Most of cellular GSH content remains in the cytosol, however it can also be found in organelles, including mitochondria, peroxisomes, endoplasmic reticulum and the nucleus [87]. Given the prominent role in keeping cells’ redox homeostasis in check, glutathione metabolism is accelerated in many types of cancer to alleviate oxidative stress and promote proliferation and metastasis [88]. High levels of GSH are associated with apoptosis-resistant phenotypes and its depletion is linked to the early stages of cell death initiation [89–91]. Nuclear and mitochondrial pool of glutathione plays an important role in protecting DNA from oxidative stress-driven lesions. Cell death induced by an intercalating drug doxorubicin was potentiated upon glutathione depletion [89]. This might serve as a rationale to design treatment and boost therapeutic effect of anticancer agents.

## NATURAL PRODUCTS DISTURBING GSH METABOLISM

Piperlongumine (PL), an alkaloid derived from long pepper was described to induce ROS in cancer but not in normal cells [92, 93]. Further studies revealed that PL treatment led to a depletion of cellular GSH and promoted ROS. The activity of chrysin and apigenin towards GSH was tested in a number of cancer cell lines, including prostate (PC-3), myeloid (HL-60) and lung (A549) cells. Both flavones proved to be effective glutathione depleting agents. Additionally, chrysin potentiated curcumin cytotoxic effect in PC-3 and HL-60 cells [94]. Doxorubicin and cisplatin cytotoxicity was also strongly induced upon chrysin treatment, which promoted GSH efflux and depletion [69, 94]. Another flavone luteolin attenuated Nrf2 signaling leading to a decreased expression of its target genes and GSH depletion in wild type mouse small intestinal cells. Luteolin sensitizied oxiplatin-resistant colorectal cancer cell lines to cisplatin, doxorubicin and oxiplatin [68] and efficiently inhibited GST leading to GSH depletion in melanoma cells [95]. Allicin, a natural compound derived from garlic, was found to induce ROS in PaCa-2 cells. Oxidative insult was concomitant with depletion of GSH, which facilitated apoptosis [96]. Plumbagin, a ROS-inducing naphthoquinone originally derived from *Plumbago* plants, was reported to cause GSH depletion and induce death of human prostate cancer cells (PC-3, LNCaP and C4-2) [97]. Phenylethyl iosothiocyanate (PEITC), naturally occurring in cruciferous vegetables, has been widely studied for its biological activity and proved to exert anti-cancer properties. PEITC strongly induced oxidative damage due to the depletion of glutathione and inhibition of GPX in H-Ras transformed ovarian epithelial cells [98]. Depletion of cellular glutathione after PEITC treatment was observed in cancer cells of different origin, including glioma, oral cavity cancer, leukemia, prostate and breast [99–103]. Recent data demonstrate that PEITC caused inhibition of GST in glioma GBM 8401cells, leading to massive ROS induction and causing cell death [104]. PEITC sensitized cancer cells to cisplatin in biliary tract through PEITC-induced depletion of overall GSH, which facilitated Mcl-1 glutathionylation, promoted Mcl-1 degradation and resensitized cells to cisplatin [105]. This data indicate that combined anticancer therapy based on synergistic effect of GSH depletion and strong oxidative stress induction leads to an effective cancer cell killing.

## THE THIOREDOXIN SYSTEM

Thioredoxin system includes thioredoxin (Trx), thioredoxin reductase (TrxR) and nicotinamide adenine dinucleotide phosphate (NADPH) (Figure 1). Thioredoxins have a conserved dithiol Cys-Gly-Pro-Cys motif in their catalytic site and participate in the reduction of oxidized proteins. Thioredoxin reductases balance the amount of reduced Trx by transferring electrons from NADPH to oxidized catalytic sites. Humans express three thioredoxin reductase isozymes: TrxR1 (cytosolic), TrxR2 (mitochondrial), TrxR3 (testis specific). Thanks to the oxidoreductase activity of thioredoxins they act as electron carriers for catalytic cycles of enzymes and protect proteins from aggregation or inactivation resulting from their oxidation [106]. Thioredoxins were described as redox regulators of a number of transcription factors like NF-ĸB, HIF1-α, VEGF, modulates matrix metalloproteinase-9 (MMP-9), therefore promoting proliferation, angiogenesis and metastasis. Apart from balancing cell redox state, Trx1 can inhibit apoptosis by binding and blocking the activity of Apoptosis Signal-Regulating Kinase 1 (ASK1), decreasing cell response to anti-cancer drugs [107–110]. Both Trx1 and TrxR1 are highly expressed in malignant cells, maintaining cell viability and protecting from apoptosis [111]. Blocking the activity of thioredoxin system lowers the cell's detoxifying potential and enhances oxidative insult. Many compounds have been studied for their activity to modulate thioredoxin system in tumor cells.

## NATURAL PRODUCTS TARGETING THIOREDOXIN SYSTEM

A study testing tea catechins for their potential to inhibit TrxR1 found that a polyphenol abundant in dried leaves of white, green and black tea, epigallocatechin gallate (EGCG), abrogated TrxR1 activity by direct targeting TrxR1 thiol groups. EGCG led to a significant decrease in HeLa cells viability [112]. EGCG anti-cancer effect was also studied in combination with luteolin in head and neck and lung cancer cell lines and in xenograft models, where they synergistically promoted p53 activation and apoptosis induction, leading to the growth inhibition and reduction of tumor volume [113]. 3-hydroxyl containing flavonoids quercetin and myricetin suppressed growth of A549 cells due to the inhibition of cellular thioredoxins. The observed effect correlated with elevated oxidized thioredoxin levels and reduced TrxR activity [114]. Pleurotin, an irreversible TrxR inhibitor displayed anti-cancer properties in MCF-7 breast cancer and HT-29 colon cancer cell lines. Inhibition of TrxR by pleurotin correlated with decreased protein levels of VEGF, HIF-1α and HIF-1α target genes in studied cell lines and in MCF-7 mouse xenografts [115]. EM23, a natural sesquiterpene lactone isolated from *Elephantopus mollis* was found to attenuate TrxR activity in CaSki and SiHa cells by direct binding to its selenocysteine site. EM23-mediated inhibition of TrxR was followed by induction of ROS and apoptosis

[116]. Chaetocin, a competitive substrate and inhibitor of TrxR, induced apoptosis in HeLa and glioma cells due to ROS induction [117, 118]. Curcumin, a polyphenol derived from *Curcuma longa* inhibited TrxR activity, leading to ROS generation in HeLa cells [107]. Javvadi et al. (2010) exploited the potential of curcumin in radiosensitization of squamous carcinoma cells. Thanks to the ability of curcumin to covalently bind to the nucleophilic residues in the C-terminal region of TrxR1, curcumin strongly inhibited its function, enhanced free radicals burst and sensitized cells to radiotherapy [119]. Clinically used inhibitor of thioredoxin reductase, auranofin, displayed synergistic lethality with GSH inhibitor piperlongumine in gastric cancer (GC) suggesting that combined inhibition of different antioxidant systems is more effective in killing cancer cells than abrogation of the activity of single ones. It again emphasizes the role of ROS scavengers as potent anticancer drug targets [120].

## SUPEROXIDE DISMUTASE

Superoxide dismutase (SOD) drives the reaction of dismutation of superoxide into hydrogen peroxide (Figure 1). There are three types of SOD in cells: CuZnSOD (SOD1) abundant in the cytosol, mitochondrial manganese superoxide dismutase MnSOD (SOD2) and extracellular ECSOD (SOD3). All superoxide dismutases carry metal ions in their active sites: SOD1 and SOD3 have zinc and copper and SOD2 carries manganese. SOD1 is mainly localized in the cytosol, but it has also been found in the outer mitochondrial membrane, where it neutralizes O_2_.^¯^ released from Complex III. SOD2 is located in the mitochondria while SOD3 remains in the extracellular matrix and prevents oxidative tissue damage [121]. MnSOD overexpression is common in tumors and contributes to therapy resistance. SOD neutralizes toxic superoxide, but as a consequence creates hydrogen peroxide, which can be further neutralized by catalase and glutathione redox cycle [122].

## NATURAL PRODUCTS BLOCKING SOD ACTIVITY

Since mitochondria are the primary source of cellular free radicals, decreasing their detoxifying ability by means of blocking SOD2 activity in tumors might contribute to the apoptosis activation. Plumbagin proved to efficiently induce apoptosis in effect in prostate cancer cell lines, partially through decreasing *SOD2* expression [97]. PEITC was found to inhibit expression of SOD in LN229 glioma cell line, weakening cellular antioxidant defense and causing apoptosis [99]. Suppression of SOD enzymatic activity by apigenin in combination with ROS-inducing paclitaxel was found to sensitize HeLa cells to apoptosis and allowed to lower paclitaxel doses [123].

## CATALASE (CAT)

Catalase is a peroxisomal enzyme that neutralizes hydrogen peroxide by its decomposition to water and oxygen (Figure 1). High levels of hydrogen peroxide facilitate DNA mutagenesis, therefore under physiological conditions catalase protects cells from oxidative damage. H_2_O_2_ also serves as mediator of apoptosis and can modify regulatory protein complexes, such as Nrf2/Keap1 system. Apart from peroxisomal CAT, malignant cells acquire membrane-associated catalase to survive under oxidative stress [124–126]. Blocking catalase activity can significantly increase oxidative burden through hydrogen peroxide accumulation which triggered tumor cells death.

## NATURAL PRODUCTS INHIBITING CATALASE

Wogonin, a flavonoid isolated from *Scutellaria baicalensis* was shown to induce cell death in cervix, ovary and lung cancer cells through catalase inhibition that increased hydrogen peroxide levels and facilitated TNF-induced apoptotic signaling [127]. Human hepatoma HepG2 cells subjected to apigenin accumulated H_2_O_2_, which correlated with a decrease of catalase mRNA and catalase activity and led to cell death [128]. PEITC treatment lowered catalase protein levels and induced ROS in GBM 8401 glioma cells [104]. EGCG inhibited catalase activity both *in vitro* and in K562 cells [129] and sensitized cells to arsenite (As) treatment. The proposed mechanism explained that the inhibition of catalase activity upon treatment with As/EGCG occurred *via* JNK (c-Jun N-terminal kinase) signaling pathway. Genotoxic stress that activated JNK, promoted catalase phosphorylation by c-Abl kinase, marking it for proteasomal degradation. Blocking catalase activity led to high amount of H_2_O_2_ and promoted death of epithelial cells subjected to As/EGCG [130].

## EXOGENOUS ANTIOXIDANTS

The role of oxidative stress in initiating and promoting cancer on the one hand and in causing oxidative damage on the other justifies two opposite ROS-manipulating strategies against cancer. First is antioxidant approach functional in cancer prevention and therapy. The most important and widespread exogenous dietary antioxidants are vitamins A and E, their analogs carotenoids and tocopherols, vitamin C and polyphenols. Though preventing ROS-induced mutations and subsequent cancer initiation with dietary antioxidants is well documented, their use during anticancer therapy remains controversial. Since cancer therapy highly relies on the production of free radicals, it has been speculated that supplying cells in antioxidants might decrease treatment efficacy. On the other hand, the basic idea behind using antioxidants during therapy is to eliminate excessive oxidative damage and to help alleviate adverse effects. Many patients receiving therapy are taking antioxidants without consulting with a physician. Selenium and vitamin C are widely used in complementary oncology [131]. Radiotherapy trials in head and neck cancers showed that vitamin E reduced the toxicity, however overall recurrence and mortality were raised [132, 133]. Trials on the effect of antioxidants on chemotherapy reported on some benefits of using vitamin E or selenium with cisplatin, taxol and oxiplatin, but the long-term effects were not assessed [134–138]. Decreased recurrence of some cancer types in patients not receiving treatment or after chemotherapy has also been reported [139, 140]. The main conclusion from these trials is that administration of antioxidants to cancer patients in combination with therapy should be taken with great care. Patient phenotype (smoking, alcohol uptake and nutrition), tumor localization (different partial pressures of oxygen among tissues) and type of therapy should be considered in order to choose a suitable antioxidant supplement [141]. Importantly, adverse effects were not reported with antioxidants derived from food. The Women's Healthy Eating and Living Study (WHELS), where diet composed of high amount of fruit and vegetable, rich in beta-carotene and vitamin C, showed no effect on outcome in patients with early breast cancer [142].

## LESSONS FROM CLINICAL TRIALS

Anticancer properties of a few natural products from Table 1 (EGCG, curcumin, resveratrol, PEITC, have been tested clinically mainly in the context of decreasing side effects caused by chemotherapy and radiation therapy or as chemopreventive dietary supplements (Table 2). These trials were basing on ROS scavenging properties of natural compounds. The ability of orally administered EGCG to reduce the incidence and severity of esophagitis was tested in patients with locally advanced stage III non-small-cell lung cancer receiving concurrent chemotherapy and thoracic radiotherapy (phase I, NCT01481818). No dose-limiting toxicity of EGCG was reported. Dramatic regression of esophagitis to grade 0/1 was observed in 22 of 24 patients and the pain score was also reduced [143]. Currently, the EGCG-mediated protection of the esophagus from damage induced by radiotherapy in patients with lung cancer is being tested in phase II (NCT02577393). Also topically administered EGCG was non-toxic and proved effective in decreasing radiation dermatitis in patients with breast cancer after mastectomy receiving adjuvant radiotherapy [144]. Orally administered curcumin significantly reduced the severity of skin reactions (dermatitis) caused by radiation therapy breast cancer patients as shown in phase II/III trial (NCT01246973) [145] and prevented colon cancer by reducing the aberrant crypt foci (ACF) number in smokers at dose 4 g/day [146]. Unfortunately, just a few trials so far addressed a question whether natural compounds could improve the efficacy of the standard chemotherapy or radiation therapy. One such a trial (phase II) tested curcumin ability to potentiate the effect of gemcitabine in patients with advanced pancreatic cancer (NCT00192842). In one out of twenty one patients evaluable for response curcumin caused brief but marked tumor regression (73%) and one patient remained stable for > 18 months. The problem was extremely limited bioavailability of curcumin as only 22 to 41 ng/mL was detectable in plasma when 8 g curcumin/day was given orally. Curcumin levels in the microgram range have been shown to be necessary to show antiproliferative effects in *in vitro* studies. Therefore, it was suggested to heat-solubilize curcumin before administration to increase its water solubility [147]. Moreover, bioactive compounds of curcumin degradation such as ferulic acid and vanillin also possess strong anticancer properties and can inhibit COX-1, COX-2 and significantly suppress NFκB activation [148–150]. In this way they may contribute to the observed biological activities of curcumin. Awaited are results of ongoing clinical trials on improved formulations of curcumin to enhance chemo- or radiotherapy (see Table 2). There is a strong need for more studies on different natural compounds as growing evidence is emerging for their benefits in improving results of standard anticancer treatments.

**Table 2:** Representative clinical trials on natural compounds modifying antioxidant response (from www.clinicaltrials.gov)

Compound/dose	Clinical trial number/phase	Purpose	Results/Status
**EGCG** 40 to 440 µmol/l 3 times a day in combination with etoposide, cisplatin, and radiotherapy	NCT01481818 Phase I	To evaluate safety and efficiency of EGCG in eosophagus protection in patients with locally advanced stage III non-small-cell lung cancer	No dose-limiting toxicity of EGCG was reported. Regression of esophagitis to grade 0/1 was observed in 22 of 24 patients at the end of radiotherapy. The pain score was reduced [143]
**EGCG** 40 to 660 μmol/l spray in the radiation field	NCT01481818 Phase I	To assess safety, tolerability and preliminary effectiveness of topical EGCG for radiation dermatitis in patients with breast cancer receiving adjuvant radiotherapy	The topical administration of EGCG was well tolerated and the maximum tolerated dose was not found. Patient-reported symptom scores were significantly decreased at 2 weeks after the end of radiotherapy in pain, burning, itching and tenderness [144]
**EGCG** 10 ml solution/day (440 µmol/l)	NCT02577393 Phase II	To evaluate the protection of the esophagus from damage induced by radiotherapy in patients with lung cancer	enrolling participants
**Polyphenon E** (PolyE, a defined green tea polyphenol extract with high EGCG content) 4 × 200 mg/day	NCT00676793 Phase II	To evaluate the short-term effects of PolyE administered during the interval between breast biopsy and surgery in women with recently diagnosed breast cancer: determination if EGCG inhibits c-Met signaling and activation of pathways contributing to breast cancer progression	completed, no results published
**Polyphenon E** 4 × 200 mg/day	NCT00676780 Phase II	To evaluate the short-term effects of PolyE administered during the interval between prostate biopsy and radical prostatectomy in men with recently diagnosed prostate cancer	A significant reduction in serum levels of prostate-specific antigen (PSA), hepatocyte growth factor (HGF) and vascular endothelial growth factor (VEGF) was observed [181]
**Polyphenon E** 2 × 200 mg/day	NCT00596011 Phase II	To determine if PolyE reduces the rate of progression to prostate cancer (PCa) in men diagnosed with high-grade prostatic intraepithelial neoplasia (HGPIN) or atypical small acinar proliferation (ASAP)	No differences in the number of prostate cancer (PCa) cases were observed but there was a decrease in a cumulative rate of progression to PCa or ASAP in a PolyE group vs. placebo group [182]
**curcumin**, 6 g/day during radiotherapy	NCT01246973 Phase II/III	To determine whether curcumin can prevent or reduce the severity of dermatitis caused by radiation therapy in breast cancer patients	Curcumin reduced the severity of radiation dermatitis in breast cancer patients [145].
**curcumin** 2 or 4 g/day for 30 days	NCT00365209 Phase IIa	To evaluate how well curcumin works in preventing colon cancer in smokers with aberrant crypt foci (ACF)	A significant 40% reduction in ACF number was observed with the 4 g dose, whereas in the 2 g group ACF were not reduced. Curcumin was well tolerated at both doses [146]
**nanostructured lipid curcumin particle** 2 × 100 mg/day	NCT02439385 Phase II	To evaluate progression-free survival in colorectal cancer patients with unresectable metastasis after treatment with Avastin/FOLFIRI in combination with a nanostructured lipid curcumin particle which improved biotransformation and bioavailability of curcumin.	This study is not yet open for participant recruitment.
**Meriva** (lecithinized curcumin delivery system) 2 × 500 mg/day	NCT01740323 Phase II	To determine if curcumin reduces NF-ĸB DNA binding in patients receiving radiotherapy for their breast cancer after having completed chemotherapy	This study is currently recruiting participants
**curcumin** 8 g/day along the chemotherapeutic protocol of weekly gemcitabine	NCT00192842 Phase II	To assess if curcumin can improve the efficacy of the standard chemotherapy gemcitabine in patients with advanced pancreatic cancer.	5 out of 17 patients (29%) discontinued curcumin due to intractable abdominal fullness or pain, and the dose of curcumin was reduced to 4 mg/day because of abdominal complaints in 2 other patients. One of 11 evaluable patients (9%) had partial response, 4 (36%) had stable disease, and 6 (55%) had tumor progression. [183]
**curcumin**, dosage not provided	NCT02095717 Phase II	To assess taxotere plus curcumin combination in first-line treatment of prostate cancer metastatic castration resistant.	study is ongoing
**nanocurcumin** SinaCurcumin^®^ 3 × 40 mg/day 3 days before and during radiotherapy	NCT02724618 Phase II	To determine the role of curcumin as a radioprotector against radiation-induced injury in normal tissues as well as a radiosensitizer in tumor in prostate cancer patients undergoing radiotherapy	recruiting participants
**curcumin capsules** dosage not provided	NCT00852332 phase II	To study how well giving docetaxel together with a curcumin works compared with giving docetaxel alone as first- or second-line therapy in treating patients with breast cancer.	recruiting participants
**Isoquercetin** 2 × 225 or 2 x: 450 mg/day along the chemotherapy with Sunitinib	NCT02446795 Phase I/II	A trial of isoquercetin as an adjunct therapy in patients with kidney cancer receiving first-line Sunitinib	This study is not yet open for participant recruitment.
**Quercetin** 2 × 250 mg / day for 3 weeks	NCT01732393	To evaluate the effect of quercetin on prevention and treatment of chemotherapy-induced oral mucositis in patients with blood malignancies.	This study has been completed, no results published
**SRT501** (micronized resveratrol) 5 g/day for 14 days	NCT00920803 Phase I	To determine safety and tolerability of SRT501 in subjects with colorectal cancer and hepatic metastases	SRT501 was well tolerated. Mean plasma resveratrol levels following a single dose of SRT501 administration were exceeding those for equivalent doses of non-micronized resveratrol by 3.6-fold. Resveratrol was detectable in hepatic tissue. Cleaved caspase-3 was significantly increased [184].
**PEITC** (dosage not provided)	NCT00691132 Phase II	PEITC in preventing lung cancer in people who smoke	The recruitment status unknown

## CONCLUSIONS

The power of natural products lies in using them as adjuvants to standard anticancer therapies but the struggle is that they often exhibit contrary actions, depending on concentration. At high doses ( > 50 µM) natural compounds presented in this article have pro-oxidant properties by limiting antioxidant capacity of cancer cells (Figure 3). Direct inhibition of cellular antioxidants or suppression of pathways leading to their expression can sensitize cancer cells to chemo- and radiotherapy. Normal cells are not that sensitive to the manipulations in redox homeostasis as their growth and proliferation are not that much ROS-dependent. Contrarily, cancer cells operate under constant oxidative stress and are very sensitive to the disruption of their enhanced ability to scavenge free radicals. Therefore, impairing antioxidant capacity of tumors emerges as a good strategy to target them. Especially inhibition of Nrf2 pathway seems a very promising approach as Nrf2 controls expression of crucial cellular antioxidants, drug efflux pumps and detoxification enzymes. Simultaneous inhibition of Nrf2 and prosurvival NFĸB signaling is even more effective in promoting death of tumor cells. Therefore, natural products that suppress Nrf2 and NFĸB pathways are promising candidates for adjuvants to chemo- and radiotherapy allowing for lowering their doses. It is nevertheless essential to bear in mind that the effect they induce in cells depends on the applied dose, cell type, exposure time and environmental conditions. The same natural product in different concentrations often possesses contrary properties. This is why it is so challenging to translate results from *in vitro* models to *in vivo* conditions. Concentrations used in cell lines experiments are often very hard to achieve in patients. Given the poor plasmatic bioavailability of active compounds and biotransformation processes they undergo in the body, the circulating concentration of natural compounds administered orally are rather low. Moreover, the biological effects they produce do not necessarily need to be a consequence of the action of only the parent compound, but might also be assigned to its metabolites. Therefore, the effects natural products present *in vivo* might be different or even opposite to expected and instead of potentiating the effect of chemo- or radiotherapy, they might weaken their action. The majority of clinical trials test ROS scavenging properties of natural compounds in the context of cancer chemoprevention or their ability to alleviate side effects of chemo- and radiotherapy. Just a few addressed a question of synergistic effects of natural products with classic anticancer therapies and the results so far warrant further investigation. There is a strong need for clinical studies testing these combination treatments in defined cancer types with special focus on bioavailability and stability of natural products.

## Abbreviations

ABCC2: ABC transporters MRP2
Akt: Protein Kinase B
AP-1: activator protein 1
ARE: antioxidant response element
As: arsenite
ASK1: apoptosis signal-regulating kinase 1
BCRP: breast cancer resistance protein
B-Raf(V619E): mutation in B-Raf that replaces amino acid valine with glutamic acid at the position 619
c-Abl: Abelson tyrosine kinase
CAT: catalase
COX1: cyclooxygenase 1
COX2: cyclooxygenase 2
Cul: cullin
EGCG: epigallocatechin gallate
EMT: epidermal to mesenchymal transition
EGFR: Epidermal growth factor receptor
ERK: extracellular signal-regulated kinase
ETC: electron transport chain
GC: gastric cancer
GSH: glutathione
GSSG: oxidized glutathione
GST: glutathione S-transferase
GST1α/β: Glutathione S-transferase 1α/β
H_2_O_2_: hydrogen peroxide
HIF-1α: Hypoxia Inducible Factor 1α
HNSCC: head and neck squamous cell carcinoma
H-Ras(G12V): mutation in H-Ras that replaces amino acid glycine with valine at the position 12
JNK: c-Jun N-terminal kinase
Keap1: Kelch-like ECH-associated protein 1
K-Ras(G12D): mutation in K-Ras that replaces amino acid glycine with aspartic acid at position 12
K-Ras: V-Ki-ras2 Kirsten rat sarcoma viral oncogene homolog
Mcl-1: Induced myeloid leukemia cell differentiation protein
MEFs: murine embryonic fibroblasts
MMP-9: matrix metalloproteinase-9
mtp53: mutant p53
Myc: myelocytomatosis oncogene
Myr-Akt: myristoylated form of Akt kinase
NADPH: nicotinamide adenine dinucleotide phosphate
NCI: National Cancer Institute
NFĸB: nuclear factor kappa-light-chain-enhancer of activated B cells
NOX: NADPH oxidases
NOX1: NADPH oxidase 1
NOX5-L: NADPH oxidase 5
NQO1: NAD(P)H-quinone oxidoreductase
Nrf2: nuclear factor (erythroid-derived 2)-like 2
O_2_.^-^: superoxide anion
OH.: hydroxyl radical
OONO-: peroxynitrite; p38
MAPK14: Mitogen Activated Protein Kinase 14
p47phox: the phagocyte NADPH oxidase/NOX2 organizer
PDPK1: 3-Phosphoinositide Dependent Protein Kinase 1
PEITC: phenylethyl iosothiocyanate
PI3K: phosphoinositide 3-kinase
PKCδ: Protein Kinase C δ
PL: piperlongumine
ROS: reactive oxygen species
SH: sulfhydryl group
SOD: superoxide dismutase
SOD1: superoxide dismutase 1
SOD2: superoxide dismutase 2
SOD3: superoxide dismutase 3
STAT3: signal transducer and activator of transcription 3
TRAIL: TNF-related apoptosis-inducing ligand
Trx: thioredoxin
Trx1: thioredoxin 1
TrxR: thioredoxin reductase
TrxR1: thioredoxin reductase 1
UGT: UDP-glucuronosyl transferases
VEGF: Vascular Endothelial Growth Factor
V-Ras: Neuroblastoma RAS viral (V-Ras) oncogene homolog
WHELS: Women's Healthy Eating and Living Study
γGCS: γ-glutamylcysteine synthetase
